# Doxycycline Enhances Anticancer Activity of Zoledronic Acid via Inducing ROS and Autophagy in Osteosarcoma Cell Lines

**DOI:** 10.7150/ijms.108086

**Published:** 2025-05-09

**Authors:** Yi-An Li, Hsuan-Ying Chen, Chien-Sheng Hsu, Shun-Cheng Tseng, Eric Hwang, Cheng-Pu Hsieh, Shih-Chieh Hung, Yi-Fu Huang

**Affiliations:** 1 Institute of Translational Medicine and New Drug Development, School of Medicine, China Medical University, Taichung, Taiwan.; 2 Department of Post-Baccalaureate Medicine, College of Medicine, National Chung Hsing University, Taichung, Taiwan.; 3 Orthopaedic Department, School of Medicine, National Yang Ming Chiao Tung University, Taipei, Taiwan.; 4 Department of Orthopedic Surgery, Changhua Christian Hospital, Changhua, Taiwan.; 5 Orthopedics & Sports Medicine Laboratory, Changhua Christian Hospital, Changhua, Taiwan.; 6 Frontier Molecular Medical Research Center in Children, Changhua Christian Children Hospital, Changhua, Taiwan.; 7 Department of Biological Science and Technology, National Yang Ming Chiao Tung University, Hsinchu, Taiwan.; 8 Institute of Molecular Medicine and Bioengineering, National Yang Ming Chiao Tung University, Hsinchu, Taiwan.; 9 Institute of Bioinformatics and Systems Biology, National Yang Ming Chiao Tung University, Hsinchu, Taiwan.; 10 Center for Intelligent Drug Systems and Smart Bio-devices (IDS2B), National Yang Ming Chiao Tung University, Hsinchu, Taiwan.; 11 Department of Kinesiology, Health and Leisure Studies, Chienkuo Technology University, Changhua, Taiwan.; 12 Drug Development Center, Institute of Translational Medicine and New Drug Development, School of Medicine, China Medical University, Taichung, Taiwan.; 13 Integrative Stem Cell Center, China Medical University Hospital, Taichung, Taiwan.; 14 Department of Orthopedics, China Medical University Hospital, Taichung, Taiwan.

**Keywords:** doxycycline, zoledronic acid, ROS, autophagy, osteosarcoma

## Abstract

Zoledronic acid (ZOL) is an inhibitor of osteoclast-mediated bone resorption. It is used to treat osteoporosis and skeletal complications in patients with tumor-induced osteolysis. ZOL is also demonstrated to possess anti-cancer activity in several tumors via apoptosis induction. Doxycycline is well-known antibiotic used in treatment of infections caused by bacteria and certain parasites. In this study, we evaluated the possibility if doxycycline could be used as an effective adjuvant to ZOL against osteosarcoma cells. The data showed that co-treatment with doxycycline at non-toxic dose could significantly increase the anti-viability effect of ZOL in osteosarcoma HOS and MG-63 cells in MTT assay and colony formation assay, and largely increased the levels of apoptotic markers, cleaved caspase 3 and PARP, in ZOL-treated cells. Furthermore, as co-treatment with doxycycline, the levels of ROS and autophagy were enhanced in ZOL-treated cells. Administration of *N*-acetyl-L-cysteine, a reactive oxygen species (ROS) inhibitor, or autophagy inhibitor chloroquine both reduced anti-growth effect of this combined treatment, indicating that the increased ROS and autophagy should be involved in anti-viability effect of combined treatment with ZOL and doxycycline. Taken together, our findings suggested that combined treatment with ZOL and doxycycline may serve as a potential strategy for treating osteosarcoma.

## Introduction

Osteosarcoma is the most frequent type of bone cancer which particularly affects rapidly growing bones in children and adolescents. It is characterized by the presence of malignant mesenchymal cells producing osteoid or immature bone. Incidence of primary osteosarcoma is about 8 cases per million per year in children and young adults 1. Surgical resection is major treatment for osteosarcoma, but the prognosis for patients who received surgery alone is poor, with a depressing 5-year survival rate of less than 20%. The introduction of adjuvant chemotherapy several decades ago improved 5-year survival rate from less than 20% to around 50% 2, 3. However, this outcome is nearly unchanged over the past decades. Therefore, identification of effective drugs or strategies are still needed for patients with osteosarcoma.

Zoledronic acid (ZOL), a third-generation nitrogen-containing bisphosphonate, is an inhibitor of osteoclast-mediated bone resorption and used to prevent or treat osteoporosis. ZOL suppresses osteoclast-mediated bone resorption through inhibiting farnesyl pyrophosphate synthase and geranylgeranyl pyrophosphate of mevalonate pathway, leading to incomplete posttranslational prenylation of small GTPases proteins including Ras, Rac, and Rho, and the absence of cholesterol. This effect ultimately induces apoptosis of osteoclasts 4.

*In vitro* studies have shown the anti-tumor activity of ZOL in various types of cancer cells including giant cell tumor, myeloma, renal cell carcinoma, breast cancer, and prostate cancer 5. ZOL directly suppresses cell proliferation and induces apoptosis in prostate and breast cancers 6-8. ZOL induces apoptosis and autophagy in cervical cancer cells 8. ZOL induces autophagic cell death in human prostate cancer cells 9. ZOL inhibits human nasopharyngeal carcinoma cell proliferation through activating mitochondrial apoptotic pathway 10. ZOL inhibits breast cancers through endoplasmic reticulum stress 11, 12.

ZOL has strong binding activity to calcified tissues. Although the half-life in plasma is less than 24 h, it is stable in bone with a half-life of more than 300 days 13. Therefore, ZOL is considered as a potential anti-cancer drug for the patients with osteosarcoma. Several studies in mice or in clinical try to demonstrate the anti-cancer activity of ZOL for osteosarcoma 14, 15. However, the outcome is still controversial. Now, some groups put the effort to improve drug sensitivity in ZOL-treated cells through combination with other treatment such as irradiation and cisplatin 16.

Doxycycline, long-acting tetracycline, is well-known antibiotic used in treatment of infections caused by bacteria and certain parasites. It blocks bacterial protein synthesis by preventing attachment of aminoacyl-tRNAs to ribosome 17. In addition, recent studies show the effect of doxycycline on anti-cancer. It inhibits mitochondrial protein synthesis and increases the levels of ROS, resulting in a slower proliferation rate in A549 lung carcinoma cell line 18. Furthermore, in a clinical study of breast cancer patients, pre-operative treatment with doxycycline results in a decrease of cancer stem cells in early breast cancer patients 19.

However, little is known about adjuvant treatment of doxycycline in cancer therapy for osteosarcoma cells. In this study, we tried to determine if doxycycline can enhance the anti-viability effect of ZOL in human osteosarcoma cell lines.

## Materials and Methods

### Cell culture and reagents

Human osteosarcoma HOS and MG-63 cell lines were purchased from the Bioresource Collection and Research Center (Hsinchu, Taiwan). HOS and MG-63 cells were cultured in Minimum Essential Medium. The cultured mediums were supplemented with 10% fetal bovine serum, 100 U/mL of penicillin/streptomycin. Zoledronic acid (ZOL) was obtained from Sigma Chemical Co. (St. Louis, MO, USA). Doxycycline were obtained as a powder from Cayman Chemical (Ann Arbor, Michigan).

### Cell viability assay

Cell viability was analyzed by MTT (3-(4,5-dimethylthiazol-2-y1)-2,5-diphenyltetrazolium bromide). HOS (3× 10^4^ cells/well) and MG-63 cells (4 × 10^4^ cells/well) were seeded in 24-well plates overnight. The cells were treated with ZOL alone, doxycycline alone, or both for 48 h. Finally, MTT was added to measure cell viability as previously mentioned 20.

### Determination of apoptosis

HOS and MG-63 cells were treated with ZOL alone, doxycycline alone, or both for 48 h. Apoptotic cells were determined by flow cytometry using the Annexin-V-FITC staining kit (Becton Dickinson, San Jose, CA, USA) according to the manufacturer's instructions 20.

### Measurement of reactive oxygen species (ROS)

HOS (1.5 × 10^5^ cells/well) and MG-63 cells (2 × 10^5^ cells/well) were seeded in 35 mm dish overnight. The cells were treated with ZOL alone, doxycycline alone, or both for 24 h. The levels of ROS in treated cells were determined by staining with DCFDA as previously mentioned 21.

### Cell Lysis and Immunoblotting

Cells were lysed in TEGN buffer (10 mM Tris, pH 7.5, 1 mM EDTA, 420 mM NaCl, 10% glycerol, and 0.5% Nonidet P-40) containing proteases inhibitor cocktail (Roche) and 1 mM dithiothreitol (DTT). For Western blotting, the cell lysates were boiled in protein sample buffer, and analyzed by SDS-polyacrylamide gel electrophoresis (PAGE). The details are described in previous report 22. The following antibodies were used: GAPDH antibody (#2118; Cell Signaling, Beverly, MA, USA), cleaved caspase-3 (#9661; Cell Signaling), PARP (#9542; Cell Signaling), LC3A/B (#12741; Cell Signaling), Beclin-1 (#3495; Cell Signaling), Atg7 (#8558; Cell Signaling).

### Colony formation assay

HOS (1.5 × 10^5^ cells/well) and MG-63 cells (2 × 10^5^ cells/well) were seeded in 35 mm dish overnight. The cells were treated with ZOL in the presence or absence of doxycycline for 24 h. The treated cells were washed twice by PBS and trypsinized, and then five hundred cells were cultured onto 35 mm dishes with drug-free complete medium for 8 to 10 days to allow colony formation. Colonies were stained by 1% crystal violet solution before counting.

### Acridine orange staining

Autophagosomes were visualized by staining with Acridine Orange (AO). The cells were grown on glass coverslips, and then treated with ZOL in the presence or absence of doxycycline for 48 h. After treatment, the coverslips were washed with PBS once and stained with AO (1 μM) for 10 min at 37 °C. AO-stained cells were washed with PBS three times, counter-stained with 4',6-diamidino-2-phenylindole (DAPI), and the cells were examined and photographed by immunofluorescence microscopy.

### Statistical analysis

All results were obtained from at least three separate experiments. Statistical comparisons of differences between groups were conducted using GraphPad Prism 4 (GraphPad Software; San Diego, CA, USA) using the Student's t-test. A *P* value less than 0.05 was considered significant.

## Results

### Cytotoxicity of zoledronic acid and doxycycline in osteosarcoma cell lines

**Figure 1:**
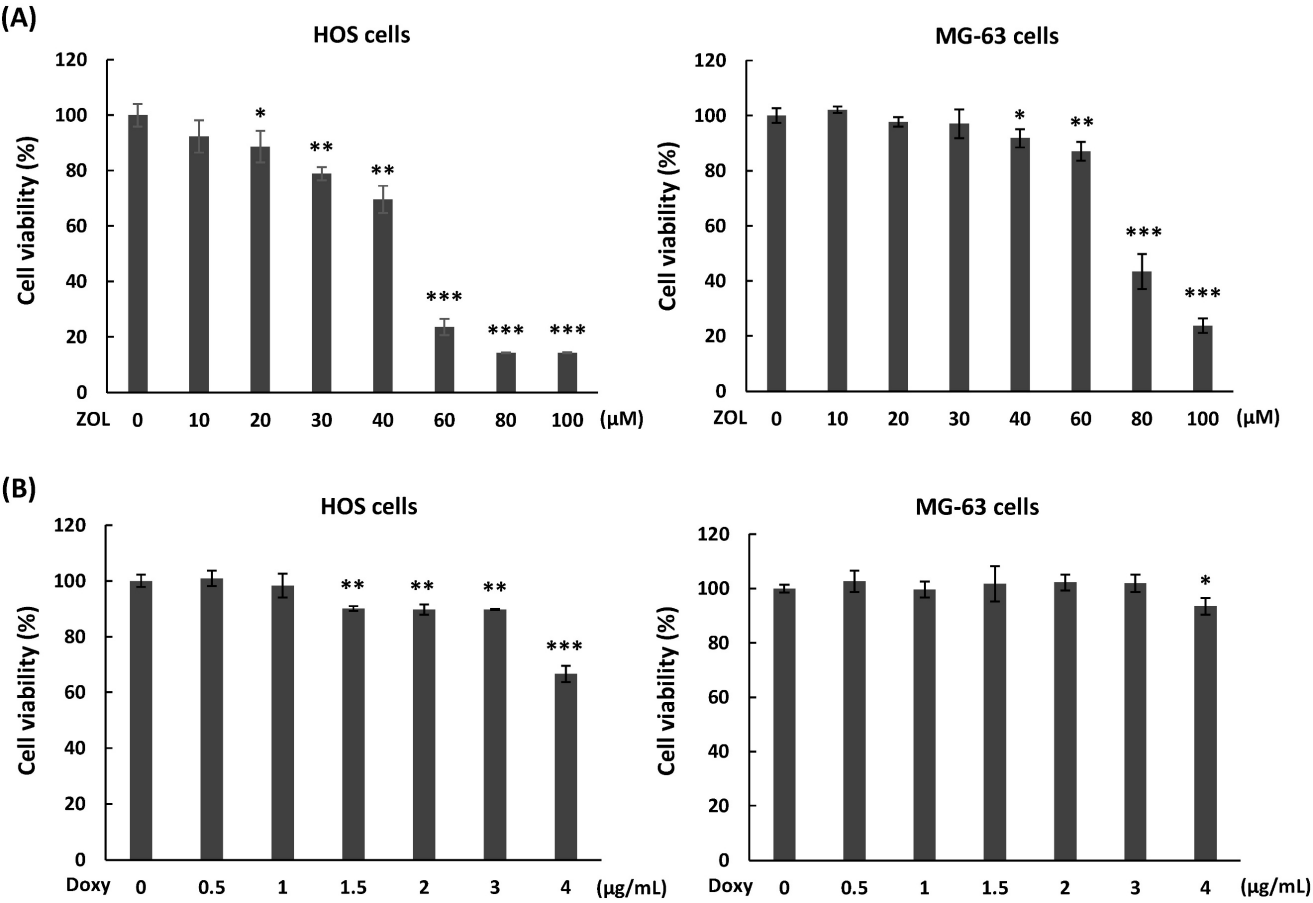
** Cytotoxic potential of zoledronic acid and doxycycline against osteosarcoma cell lines.** HOS and MG-63 cells were exposed to different doses of zoledronic acid (ZOL) **(A)** or doxycycline (Doxy)** (B)** for 48 h. MTT assay were performed to analyze cell viability. Statistical significance was determined by student *t*-test. (*) *P* <0.05, (**) *P* <0.01, and (***) *P* <0.001 as compared with 0 μM group.

To determine the effects of zoledronic acid (ZOL) on cell viability in osteosarcoma cell lines, two human osteosarcoma cell lines, one from a female patient (HOS) and one from a male patient (MG-63), were treated with different concentration of ZOL (10 to 100 μM) for 48 h, and cell viability was analyzed by MTT assay. The results showed that 60 to 100 μM of ZOL in HOS cells and 80 to 100 μM of ZOL in MG-63 cells caused obvious suppression of cell viability on these cells (Figure 1A). The IC50 (producing half-maximal inhibition) for HOS cells was approximately 52 µM at 48 h, and the concentration for MG-63 cells was approximately 75 μM at 48 h. Next, we examined cytotoxic effects of doxycycline on osteosarcoma cell lines. As shown in Figure 1B, concentrations of doxycycline less than 1 μg/mL in HOS cells and 3 μg/mL in MG-63 cells did not significantly decrease cell viability after a 48-h treatment.

### Co-treatment with doxycycline enhances anti-viability effect of zoledronic acid in osteosarcoma cell lines

**Figure 2:**
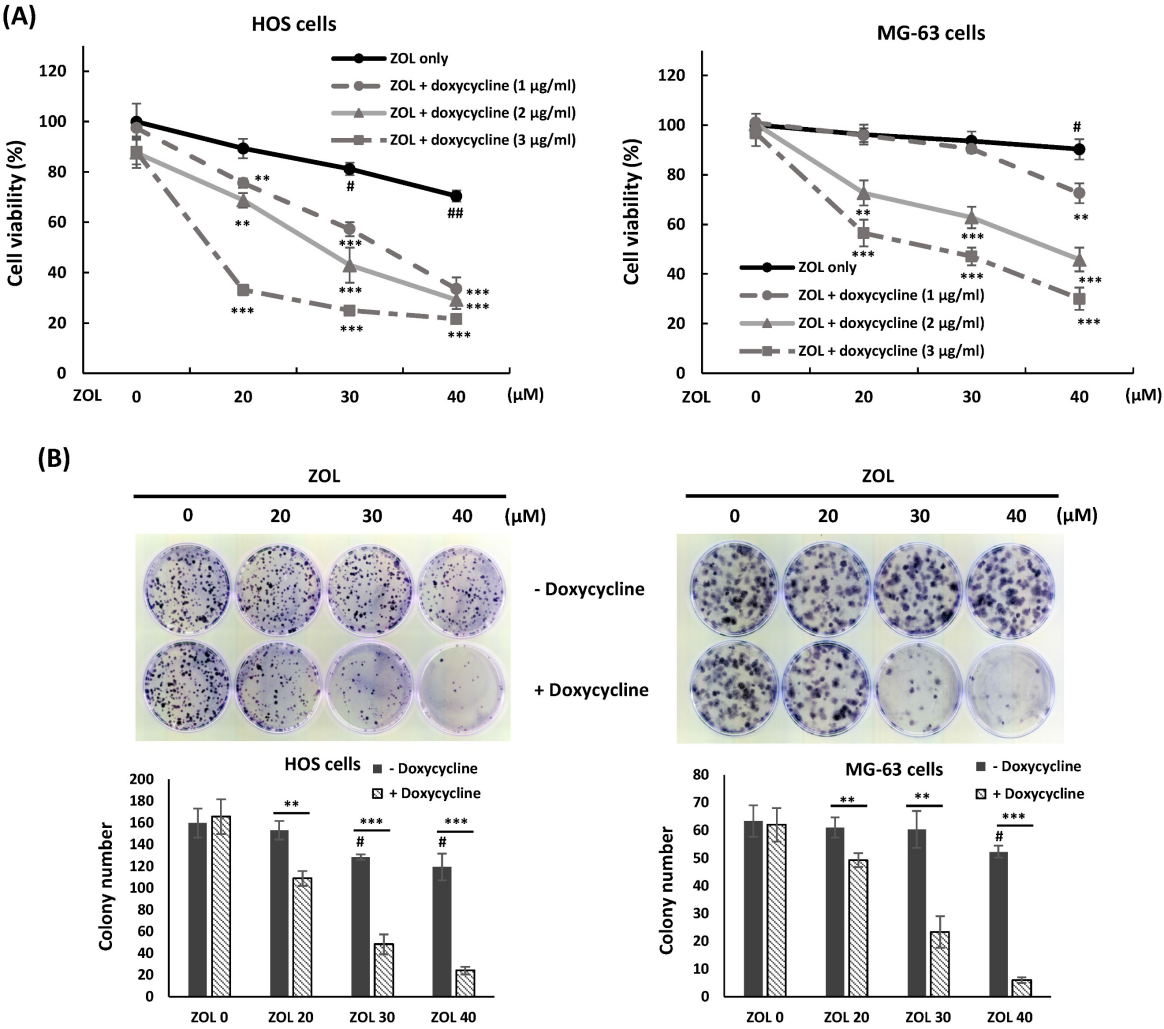
** Doxycycline enhances anti-viability effect of zoledronic acid in osteosarcoma cell lines. (A)** Co-treatment with doxycycline further inhibits cell viability in zoledronic acid-treated cells. HOS and MG-63 cells were exposed to zoledronic acid (ZOL) in the presence or absence of doxycycline for 48 h. MTT assay were performed to analyze cell viability. **(B)** Co-treatment with doxycycline further suppresses single-cell proliferative activity in zoledronic acid-treated cells. HOS and MG-63 cells were exposed to ZOL in the presence or absence of doxycycline for 24 h. The treated cells were washed by PBS and trypsinized, and then 500 cells were seeded and cultured in 35 mm dish with drug-free medium for 8 to 10 days. The colonies were stained by crystal violet and counted. The data were representative of three separately experiments and were shown as mean ± S.E.M. Statistical significance was determined by student *t*-test. (#) *P* < 0.05 and (##) *P* < 0.01 as compared with untreated group. (**) *P* < 0.01 and (***) *P* < 0.001 as compared with the single treatment with ZOL.

To investigate if co-treatment with doxycycline enhances cytotoxic effect of ZOL, HOS or MG-63 cells were exposed to ZOL (20, 30, or 40 μM) in the presence or absence of doxycycline (1, 2, or 3 μg/mL) for 48 h, and cell viability was determined by MTT assay. The results showed that ZOL treatment alone at the indicated concentrations just slightly suppressed cell viability, but co-treatment with doxycycline enhanced this suppression in these cells (Figure 2A). In addition, the combined treatment with ZOL and doxycycline resulted in a further decrease of colony-forming activity compared to ZOL alone in colony formation assay (Figure 2B).

**Figure 3:**
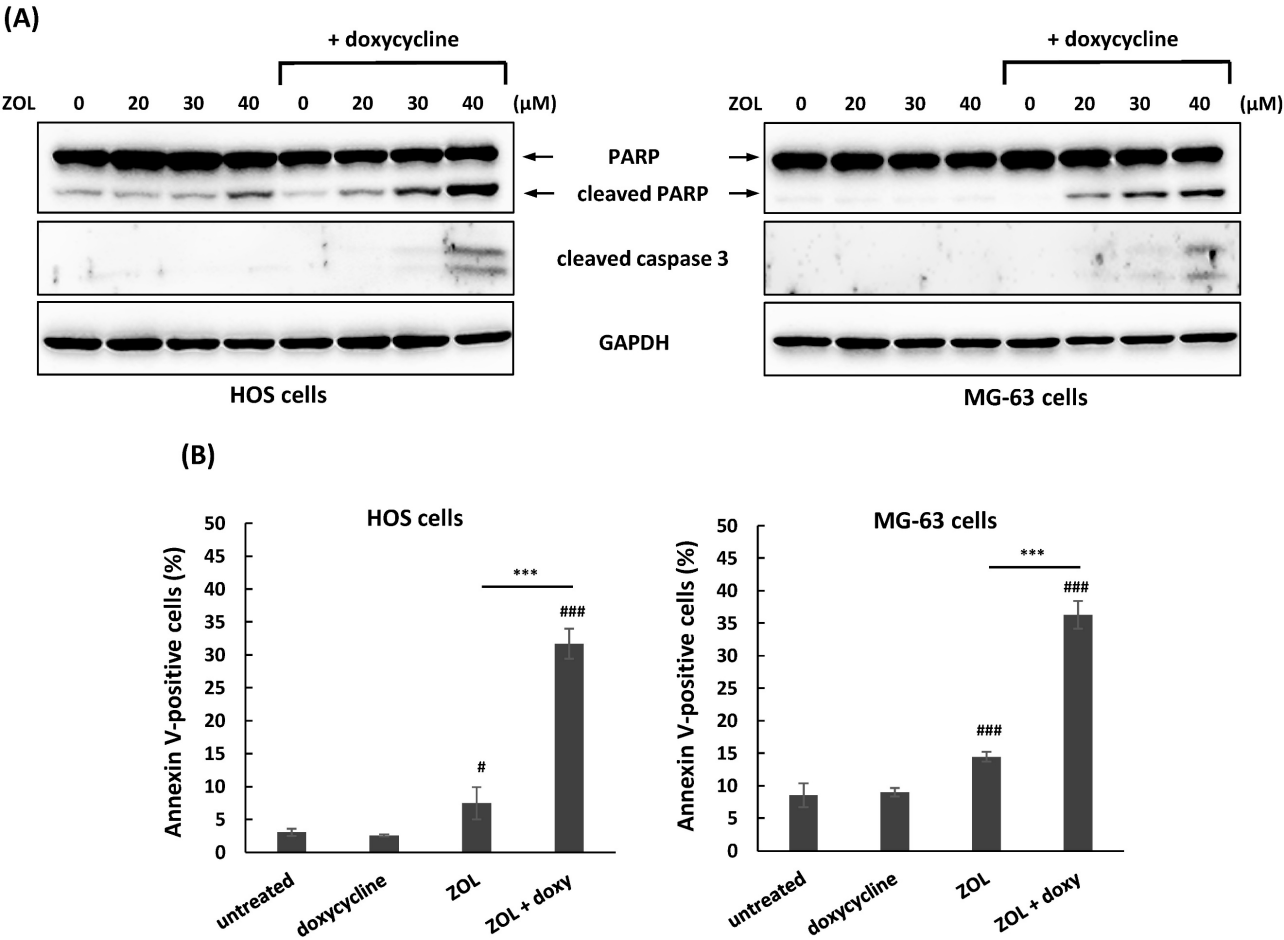
** Co-treatment with doxycycline enhances apoptosis in zoledronic acid-treated osteosarcoma cell lines.** HOS and MG-63 cells were exposed to zoledronic acid (ZOL) in the presence or absence of doxycycline (doxy) for 48 h. To detect apoptotic cells in treated cells, expression of apoptosis-related proteins was measured by Western blotting (A). And, HOS and MG-63 cells were exposed to ZOL (40 μM) in the presence or absence of doxy for 48h. The treated cells were stained with Annexin V and PI, and analyzed using flow cytometry. Quantitative data of Annexin V-positive cells are revealed (B). Statistical significance was determined by student *t*-test. (#) *P* < 0.05, and (###) *P* < 0.001 as compared with untreated group. (****) *P* < .0001 as compared with the single treatment with ZOL.

Apoptosis is involved in anti-growth effect of ZOL in several cancer cell lines 5. To investigate if co-treatment with doxycycline influences ZOL-mediated apoptosis in osteosarcoma cell lines, the levels of apoptosis-related markers were determined by Western blotting in these cells. A non-toxic concentration of doxycycline (1 and 3 μg/mL in HOS and MG-63 cells, respectively) that determined by MTT assay (Figure 1B) was used for continued combined treatment. The data showed that co-treatment with doxycycline resulted in the enhanced levels of apoptosis (cleaved PARP and cleaved caspase 3) in ZOL-treated cells compared to ZOL alone (Figure 3A). Furthermore, this combined treatment with doxycycline and ZOL significantly increased the rate of Annexin-positive cells compared to single-drug treatment (Figure 3B). These results indicated that co-treatment with doxycycline enhanced anti-growth of ZOL in osteosarcoma cell lines, and the up-regulation of apoptosis should be involved in this effect.

### Increased reactive oxygen species (ROS) and autophagy by co-treatment of doxycycline enhance anti-viability of zoledronic acid

**Figure 4:**
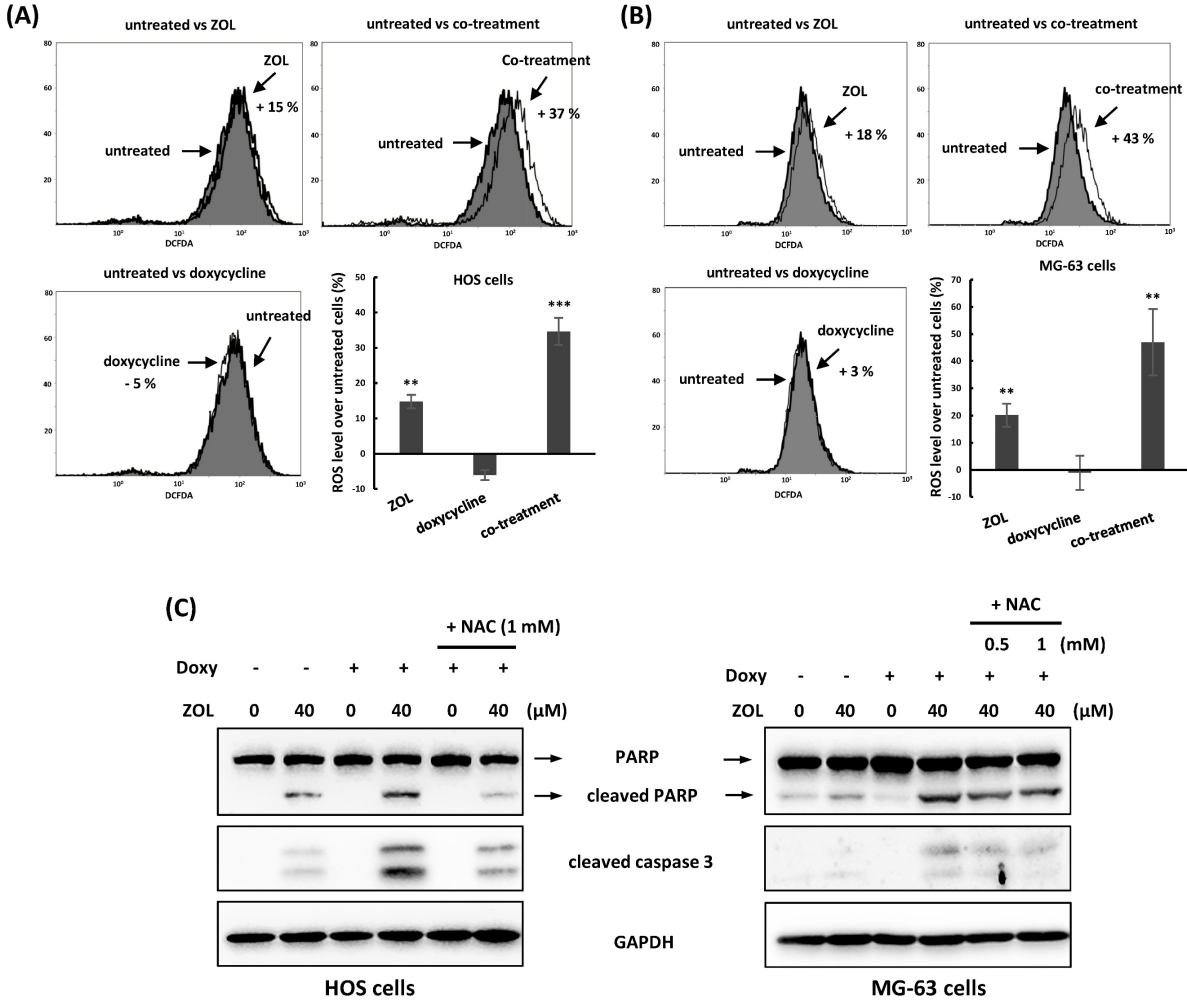
** Reactive oxygen species (ROS) are involved in the apoptosis that triggered by combined treatment with zoledronic acid and doxycycline. (A)** Co-treatment with doxycycline increases ROS generation in zoledronic acid-treated cells. HOS and MG-63 cells were exposed to either zoledronic acid (ZOL) (40 μM), doxycycline or a combination of ZOL/doxycycline for 24 h. The treated cells were stained with 2',7'-dichlorofluorescin diacetate (DCFDA), and ROS levels were determined by flow cytometry. Numbers indicated the induced levels of ROS in drug-treated cells compared to untreated cells. The results were calculated by a median of fluorescence intensity in each group. **(B)** *N*-acetyl-L-cysteine (NAC) suppresses the apoptosis induced by combined treatment with zoledronic acid and doxycycline. HOS and MG-63 cells were exposed to either zoledronic acid (ZOL) alone, doxycycline (Doxy) alone or both with or without NAC for 48 h. The levels of cleaved PARP and caspase 3 were determined by Western blotting in treated cells. Statistical significance was determined by student *t*-test. (**) *P* <0.01 and (***) *P* <0.001 as compared with untreated group.

Several chemotherapy drugs including ZOL have the ability to induce reactive oxygen species (ROS) generation and then suppressed cell viability in cancer cells 23. To determine the roles of ROS in combined treatment with ZOL and doxycycline in osteosarcoma cell lines, 2',7'-dichlorofluorescin diacetate (DCFDA), a cell-penetrable ROS sensor dye, was used to measure ROS generation in these cells. When DCFDA is oxidized by ROS, it produces a green fluorescent compound called 2,7-dichlorofluoroscein. Our results showed as the cells were treated with ZOL alone for 24 h, the levels of ROS were increased in these cells compared with untreated cells. Importantly, treatment with doxycycline alone for 24 h didn't influence ROS generation in cells, but co-treatment with this drug significantly increased the levels of ROS in ZOL-treated cells (Figure 4A and 4B).

**Figure 5:**
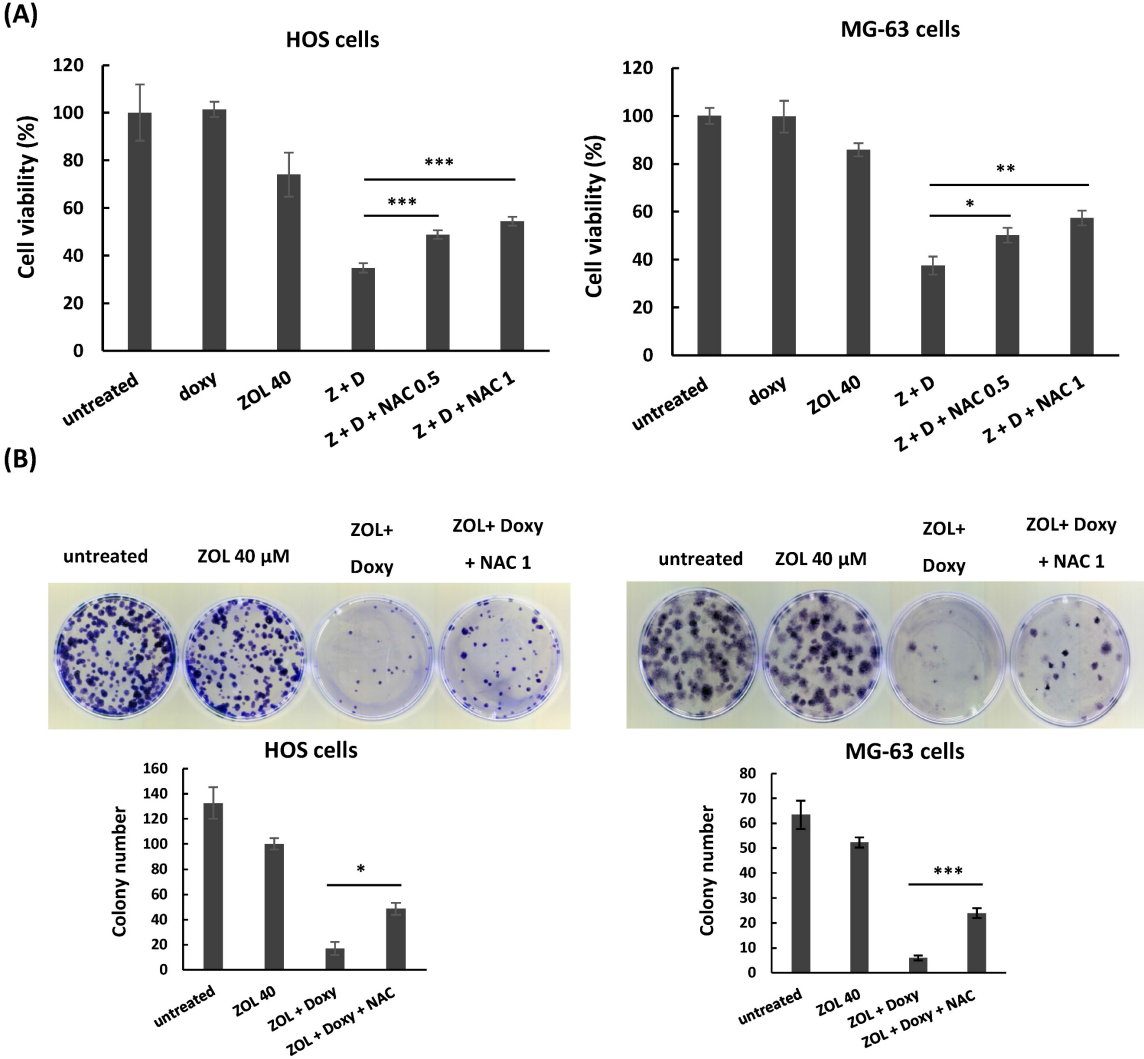
***N*-acetyl-L-cysteine (NAC) rescues anti-viability effect of combined treatment with zoledronic acid and doxycycline in osteosarcoma cell lines**. HOS and MG-63 cells were exposed to either zoledronic acid (ZOL) alone, doxycycline (doxy or D) alone or both with or without NAC (0.5 or 1 mM). Cell viability was determined by MTT assay **(A)**, and clonogenic survival was analyzed by colony formation assay in treated cells **(B)**. Statistical significance was determined by student *t*-test. (*) *P* <0.05, (**) *P* <0.01, and (***) *P* <0.001 as compared with combined treatment with ZOL and doxycycline.

Furthermore, administration of NAC (N-acetyl-l-cysteine), a ROS inhibitor, suppressed the increased cleaved PARP and caspases 3 (Figure 4C), and reversed the suppressed cell viability (Figure 5A) and colony-forming ability (Figure 5B) in the cells with combined treatment. These results indicate that combined treatment with ZOL and doxycycline largely enhance ROS generation, and this ROS generation should be involved in anti-viability effect of the combined treatment in osteosarcoma cell lines.

**Figure 6:**
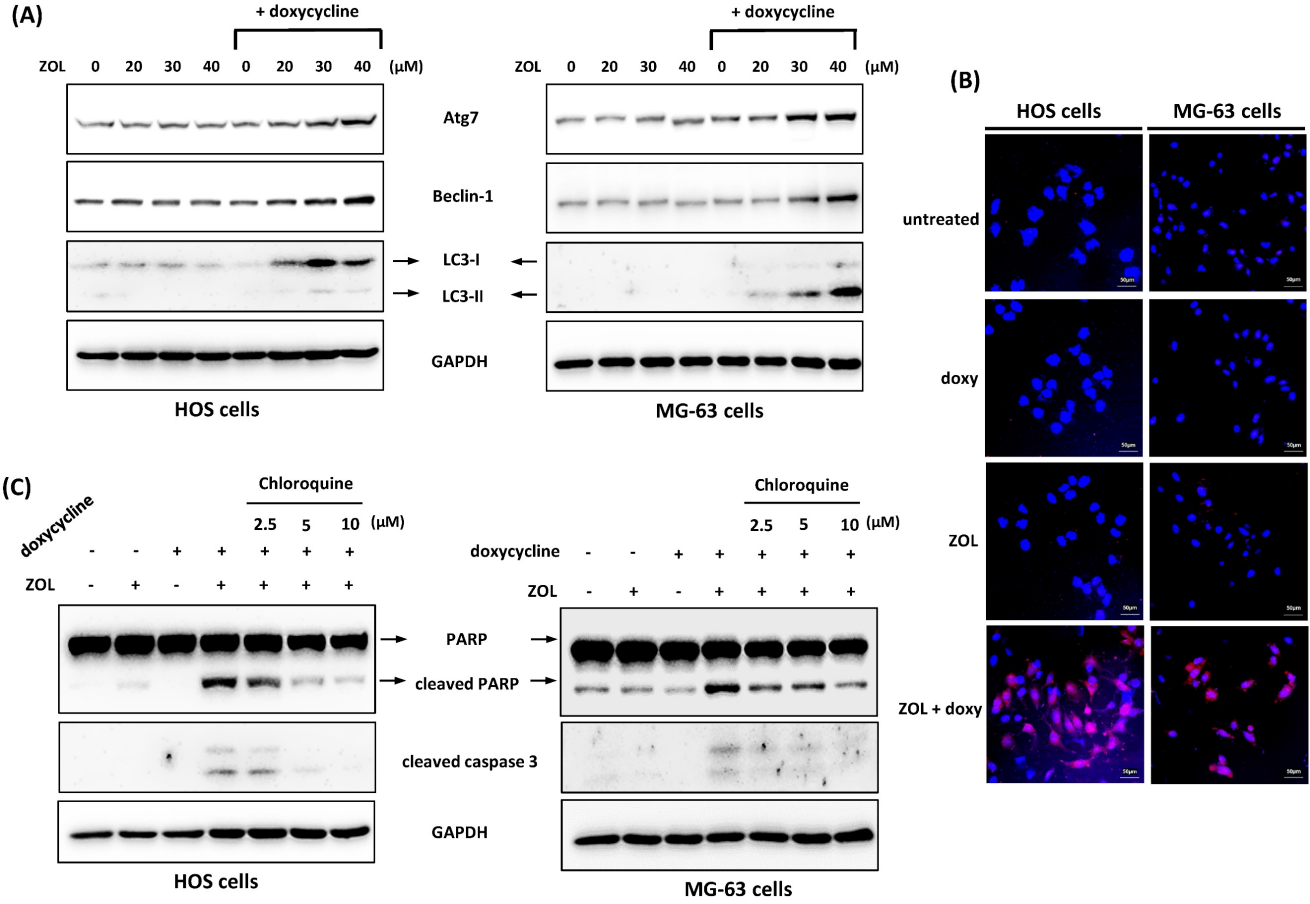
** Autophagy is involved in anti-viability effect of combined treatment with zoledronic acid and doxycycline in osteosarcoma cell lines. (A)** Co-treatment with doxycycline enhances autophagy in zoledronic acid-treated cells. HOS and MG-63 cells were exposed to zoledronic acid (ZOL) in the presence or absence of doxycycline for 48 h. The level of autophagy-related protein was determined by Western blotting in treated cells. **(B)** Zoledronic acid combines with doxycycline to induce acidic autophagic vacuoles. The cells were treated with either doxycycline alone (doxy), zoledronic acid (ZOL) alone (40 μM), or both (ZOL+doxy) for 48 h. The cells were stained with acridine orange (AO) to observe acidic (autophagic) vacuoles (red color), and 4',6-diamidino-2-phenylindole (DAPI) for labeling nucleus (blue color). **(C)** Chloroquine suppresses the apoptosis induced by combined treatment with zoledronic acid and doxycycline. HOS and MG-63 cells were exposed to either zoledronic acid (ZOL) (40 μM) alone, doxycycline alone, or both in the presence or absence of chloroquine for 48 h. The levels of cleaved PARP and caspase 3 were determined by Western blotting in treated cells.

We also tested other mechanisms or pathways that potentially involved in the combined effect including autophagy. Autophagy is a cellular process used to recycle or degrade proteins and cytoplasmic organelles in response to stress. Its accumulation is shown to be associated with antitumor effect of several chemotherapy drugs 24. Our results showed that co-treatment with doxycycline increased the level of LC3-II, the marker protein for autophagy, in ZOL-treated cells. We also determined the expression of Atg7 and Beclin-1, two proteins known to be crucial for the execution of autophagy. Similar to LC3-II expression, Atg7 and Beclin-1 levels were increased by combined treatment with ZOL and doxycycline (Figure 6A). In addition, by acridine orange staining, an enhanced formation of autophagic vacuoles as appeared as red fluorescent was observed in cells exposed to both ZOL and doxycycline (Figure 6B). These data indicate that autophagy could be enhanced by this combined treatment.

**Figure 7:**
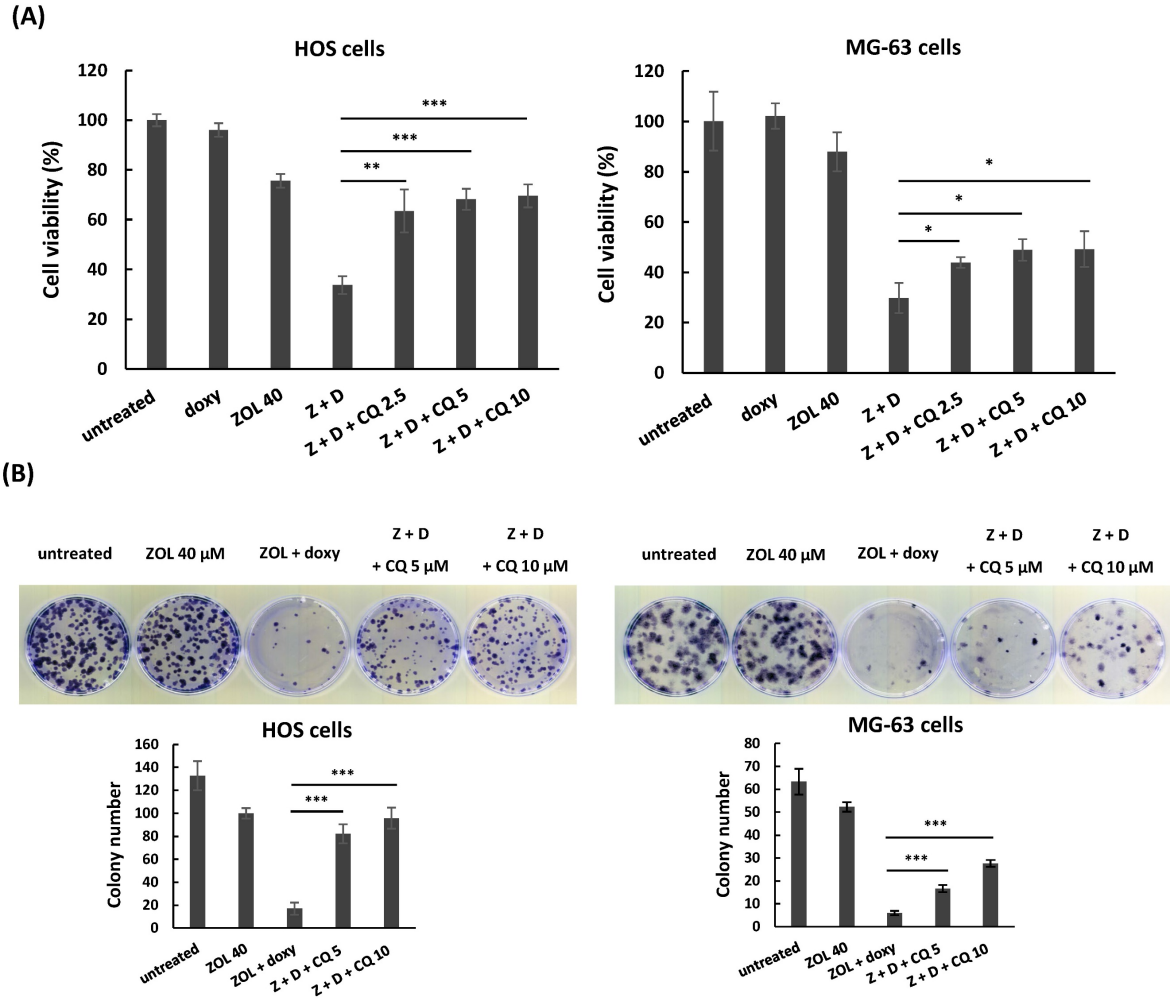
** Chloroquine attenuates anti-viability effect of combined treatment with zoledronic acid and doxycycline in osteosarcoma cell lines.** HOS and MG-63 cells were exposed to either zoledronic acid (ZOL) alone, doxycycline (doxy or D) alone, or both in the presence or absence of chloroquine (CQ) (2.5, 5, or 10 μM). Cell viability was determined by MTT assay **(A)**, and clonogenic survival was analyzed by colony formation assay in treated cells **(B)**. Statistical significance was determined by student *t*-test. (*) *P* <0.05, (**) *P* <0.01, and (***) *P* <0.001 as compared with combined treatment with ZOL and doxycycline.

Next, we used chloroquine, an autophagy inhibitor, to further analyze the role of autophagy in anti-viability effect of this combined treatment. The data showed once the autophagy was blocked by chloroquine, the increased levels of cleaved caspase 3 and PARP in cells with combined treatment were reduced (Figure 6C), and the suppressed cell viability and colony-forming ability were restored in these cells (Figure 7A and 7B). These results indicate that the enhanced autophagy should be involved in anti-viability effects of the combined treatment in osteosarcoma cell lines.

## Discussions

Current trends in the treatment of human tumors are with drug combinations that could provide a better response as well as minimize the used concentrations of the drugs. Literatures show that zoledronic acid (ZOL) could be combined with some chemotherapeutic agents to result in synergistic suppression on cancer cell activity. For example, the combined use of ZOL could increase cytotoxic effects of paclitaxel, cisplatin, or doxorubicin in breast cancer cells 25-27. In this report, we demonstrated that cytotoxic effects of ZOL were enhanced by doxycycline in osteosarcoma cells. The results showed that co-treatment with doxycycline further suppressed cell viability and increased apoptotic cells in ZOL-treated cells via the generation of reactive oxygen species (ROS) and autophagy.

ROS is considered as a potential target for cancer therapy 28. Compared to normal cells, cancer cells exhibit higher levels of ROS due to metabolic reprogramming. Increased ROS play crucial roles in cancer formation and progression. However, once ROS levels are pushed over the cytotoxic threshold, cancer cells will be killed. Several widely used chemotherapeutic agents and radiation therapy rely on ROS accumulation as a mechanism to trigger cancer cell death 29, 30. In this report, our results showed that ZOL treatment alone at a dose of 40 μM slightly increased ROS levels, but this ROS levels could be amplified by co-treatment with doxycycline followed by huge apoptotic cell death in ZOL-treated cells. It seems that the amplified ROS have reached the threshold to kill cancer cells.

However, it is uncertain how co-treatment with doxycycline affect ROS production in ZOL-treated cells. Dijk et al. have demonstrated the effect of doxycycline on mitochondria, the major site of ROS production and elimination, in lung adenocarcinoma cell line A549. Their results show that treatment with doxycycline (10 μg/mL) for five days results in a decrease of mitochondrial protein synthesis and mitochondrial membrane potential, and an increase of ROS, and slow proliferation and have no effect on apoptosis in A549 cells. This treatment also decreases the levels of glutathione, the main cellular antioxidant, in A549 cells 18.

In our report, treatment with doxycycline alone (1 and 3 μg/mL in HOS and MG-63 cells, respectively) for 24 h didn't increase ROS levels, and for 48 h didn't affect cell viability in osteosarcoma cell lines. However, co-treatment with doxycycline in ZOL-treated cells resulted in a further increase of ROS and decrease of cell viability compared with ZOL alone. Based on these findings, we reasoned that treatment with doxycycline could cause an attenuation of mitochondrial function. This attenuation may not influence cell proliferation or viability in a short period of time. However, it could result in the loss of tolerance to ROS accumulation, and then the cells become more sensitive to some ROS inducers such as ZOL or polyphyllin G as mentioned before 18.

Autophagy is a lysosome-dependent self-digestive program to recycle damaged proteins and organelles in cells. It plays a dual role of either pro-cell survival or pro-cell death in response to different chemotherapy. For example, autophagy induced by cisplatin protect cells from apoptosis in ovarian cancer cell line A2780 31. However, autophagy activated by combination of cisplatin and resveratrol, one of natural phytoalexin products, result in a decrease of cell viability in lung carcinoma cell line A549 32. In this report, the data showed that co-treatment with doxycycline resulted in an increased level of autophagy in ZOL-treated osteosarcoma cell lines. Suppression of this autophagy by chloroquine decreased inhibitory effect of the combined treatment on cell viability and proliferation in these cells, indicating that this induced autophagy should work as a pro-death role in the combined treatment.

It is known that autophagy and ROS can regulate each other in some chemotherapy. For example, Khandelwal et al. show that increased ROS is essential for the induction of autophagy in ZOL-treated breast cancer cell line MCF-7 33. In this report, we found that both ROS and autophagy could be contributed to anti-viability of the combined treatment, but their interaction should be addressed further.

Two human osteosarcoma cell lines, one from a female patient (HOS) and one from a male patient (MG-63), were used in this study. As the cells were exposed to the combined treatment of ZOL and doxycycline, cell viability was suppressed, and the increased ROS and autophagy followed by apoptotic cell death were observed in both cell lines. And, autophagy-related proteins, Atg7 and Beclin-1, were increased in both cell lines (Figure 6A). However, Atg3 was enhanced in HOS cells, but not in MG-63 cells (data not shown), suggesting that there are some differences in autophagy formation between HOS and MG-63 cells in response to this combined treatment.

## Conclusion

In this report, we demonstrated that doxycycline works as a chemosensitizer to enhance anti-viability effect of zoledronic acid via apoptosis in osteosarcoma cell lines. The increased ROS and autophagy should be involved in this process. Our findings suggested that this combined treatment may serve as a potential strategy for treating osteosarcoma.
